# Cell Culture Based *in vitro* Test Systems for Anticancer Drug Screening

**DOI:** 10.3389/fbioe.2020.00322

**Published:** 2020-04-09

**Authors:** Kristina V. Kitaeva, Catrin S. Rutland, Albert A. Rizvanov, Valeriya V. Solovyeva

**Affiliations:** ^1^Institute of Fundamental Medicine and Biology, Kazan Federal University, Kazan, Russia; ^2^School of Veterinary Medicine and Science, University of Nottingham, Nottingham, United Kingdom

**Keywords:** drug screening, two-dimensional cultures, three-dimensional cultures, microfluidic systems, Boyden chamber, tumor microenvironment, 3D bioprinting

## Abstract

The development of new high-tech systems for screening anticancer drugs is one of the main problems of preclinical screening. Poor correlation between preclinical *in vitro* and *in vivo* data with clinical trials remains a major concern. The choice of the correct tumor model at the stage of *in vitro* testing provides reduction in both financial and time costs during later stages due to the timely screening of ineffective agents. In view of the growing incidence of oncology, increasing the pace of the creation, development and testing of new antitumor agents, the improvement and expansion of new high-tech systems for preclinical *in vitro* screening is becoming very important. The pharmaceutical industry presently relies on several widely used *in vitro* models, including two-dimensional models, three-dimensional models, microfluidic systems, Boyden’s chamber and models created using 3D bioprinting. This review outlines and describes these tumor models including their use in research, in addition to their characteristics. This review therefore gives an insight into *in vitro* based testing which is of interest to researchers and clinicians from differing fields including pharmacy, preclinical studies and cell biology.

## Introduction

The number of patients diagnosed with cancer is increasing worldwide and one of the most important challenges remains the development of effective, safe and economically viable antitumor drugs. Clinical approval for drugs tested in preclinical studies enabling them to enter phase I clinical trials is essential. Currently, potential anticancer drugs have a very low rate of gaining clinical approval at around 7%, much lower than drugs for other diseases (Hay et al., 2014). Given the high cost and duration of anticancer drug clinical development it is necessary to develop new, more effective preclinical platforms for screening antitumor compounds (Imamura et al., 2015).

*In vitro* tumor models are a necessary tool in not only the search for new substances showing antitumor activity but additionally for assessing their effectiveness. Realistic *in vitro* models of tumors enable more detailed primary screening of potential antitumor drugs thus preventing drugs with insufficient antitumor activity from entering preclinical animal testing. Pharmacological testing on animal models is carried out to assess bioavailability, toxicity at specific doses and therapeutic efficacy of compounds (Stevens and Baker, 2009). According to industry standards, any novel drugs must undergo preclinical trials using animal models before being admitted to human clinical trials. However, the use of animal models can cause a number of problems including high cost, differential responses due to physiological variations between species, and limitations in test availability and feasibility (Bileckot et al., 1991). This presents an opportunity and a requirement for the creation of more high-tech *in vitro* models to assess the therapeutic efficacy of antitumor drugs.

## Tumor Microenvironment

The behavior of the tumor in the body is determined by cells within the tumor and stromal tumor microenvironment (TME) and the extracellular matrix (ECM), which provides structural support for cells in the extracellular space (Chiantore et al., 2015). The TME is characterized by a low extracellular pH and a high level of hypoxia, both factors moderate dormant phenotypes of tumor cells. As a result, these factors are associated with development of therapy resistance and poor prognosis of tumor-bearing patients (Peppicelli et al., 2017; Butturini et al., 2019). The tumor biological characteristics are similar to the chronically unhealed wound with constant inflammation, which contributes toward tumorigenesis, tumor progression and metastasis (Gal et al., 2017). Attracted by the tumor stromal microenvironment, other cell types also play a key role in not only tumor progression and metastasis, but also in the formation of resistance to therapies (Wu and Dai, 2017). Within the TME many other cellular components reside including immune cells (T-lymphocytes, B-lymphocytes, neutrophils, natural killer cells (NK-cells) and macrophages), endothelial cells associated with the tumor, fibroblasts, myofibroblasts, adipocytes, pericytes and mesenchymal stem/stromal cells (MSCs) (Chiantore et al., 2015).

The stromal cells and fibroblasts within the TME are known to secrete growth factors and chemokines, which support the growth and survival of malignant cells and additionally function as chemoattractants that stimulate the migration of other cells into the tumor (Hanahan and Coussens, 2012). MSCs are involved throughout every stage of tumor development: avoiding immunological surveillance, stimulating tumor angiogenesis, developing resistance to therapy, invasion and metastasis, as well as inducing the transition of tumor cells into a low-differentiated state and the formation of stem tumor cells (Sun et al., 2014). Of great interest is the interaction between immune cells and tumor cells, this is primarily due to the dual role of immune cells and the factors they produce. Immune reactions prevent and inhibit the development of tumors, however, recent evidence suggests that immune cells in the tumor microenvironment closely interact with transformed malignant cells, thus promoting oncogenesis (Payne et al., 2014).

An important component of TME is the ECM, consisting of components with various physical and biochemical properties, including proteins, glycoproteins, proteoglycans and polysaccharides (Insua-Rodriguez and Oskarsson, 2016). ECM provides physical support for TME cells, and also it is a source of key growth factors. In the late stages of the ECM become disorganized. ECM modulates the behavior of stromal cells in the tumor microenvironment, which leads to the induction of inflammatory reactions and the growth of new blood vessels (Trivanovic et al., 2016).

Thus, the study of the tumor as a complex environment can make a significant contribution to improving the quality of cancer treatment, as can the development of new diagnosis and personalized therapeutic methodologies (Chulpanova et al., 2018a,b,c), alongside the creation of new, realistic tumor models for the effective screening of new substances exhibiting potential antitumor activity.

## Two-Dimensional Cultures

Until the 1980s, the National Cancer Institute (NCI) used *in vivo* mouse models of P388 or L1210V leukemia for systematic screening of drugs (Waud, 2011). These models possessed high levels of productivity and stability, were convenient for data interpretation, and were relatively inexpensive. Despite these qualities, a significant drawback to these models was the inability to identify potential antitumor substances aimed at treating solid tumors. This drawback was taken into account, and by the end of the 80s, an *in vitro* panel for drug screening was developed, consisting of 60 different human cell lines originating from tumors (leukemia, melanoma, tumors of the central nervous system, cancer of the lungs, colon, ovaries, breast, kidney, and prostate), which was called NCI60 (Mingaleeva et al., 2013).

Testing a drug of interest using the NCI60 panel involves the application of two-dimensional (2D) tumor cell cultures, grown in a monolayer on a flat surface (Takimoto, 2003). During the first stage of screening, testing is carried out on the three cell lines that are frequently the most sensitive to drug therapy, MCF7 (breast adenocarcinoma), NCI-H460 (lung carcinoma) and SF-268 (glioma) (Blatt et al., 2013). The cytotoxicity of the test substance is determined using the pink anionic dye sulforodamine B. If the test substance inhibits the growth of at least one cell line, testing proceeds to the next stage comprising of the full 60 cell line panel (Mingaleeva et al., 2013). In 2017, the NCI ALMANAC database was created based on screening results using the NCI60 panel^1^. The database helped identify new effective combinations of existing antitumor drugs and new clinical trials were launched (Holbeck et al., 2017).

**FIGURE 1:**
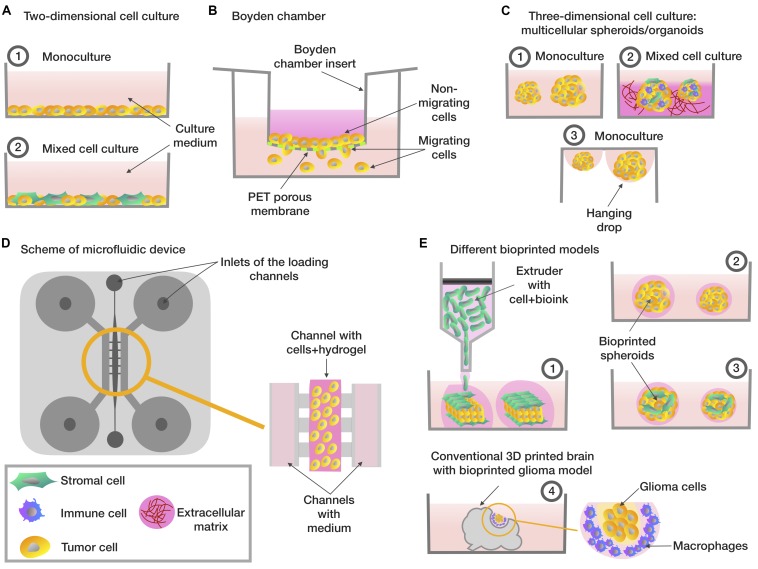
Different types of *in vitro* tumor models. **(A)** Two-dimensional cell cultures based on monolayer (1) consisting of tumor cells **(2)** co-culture included tumor and stromal cells. **(B)** The Boyden chamber scheme, analyzing the ability of cells to migrate – cells with high invasive potential pass through the porous membrane. **(C)** Three-dimensional cellular models based on multicellular spheroids/organoids: (1) spheroids consisting of tumor cells **(2)** a tumor stroma model based on the co-cultivation of several types of cells on extracellular matrix model model or on the organoid-based manner **(3)** spheroids created using the hanging drops method. **(D)** Scheme of microfluidic system that evaluates the invasive potential of tumor cells – a mixture of hydrogel and cells is placed in the central channel, into the lateral channels placed the enriched/depleted factors medium depending on the purpose of the experiment. **(E)** Tumor bioprinting models (1) a tumor model, which is a layer of tumor cells located between the layers of stromal cells **(2)** bioprinted spheroids consisting of tumor cells **(3)** bioprinted spheroids, which are a model of the tumor stroma, consisting of tumor cells mixed with stromal cells **(4)** a glioma model, consisting of conventional 3D-printed model of the brain with glioma cells and macrophages embedded in.

By analogy with the NCI60 panel, the Japanese Foundation for Cancer Research (JFCR) developed a panel in the 1990s consisting of 30 tumor lines from the NCI60 panel, plus nine tumor cells lines specific to the Japanese population, specifically gastric cancer cells (St-4, MKN-1, MKN-7, MKN-28, MKN-45, and MKN-74) and breast cancer cells (HBC-4, HBC-5, and BSY-1). Thus, the panel included 39 cell lines and was therefore called JFCR39 (Nakatsu et al., 2007). However, during clinical trials, it became apparent that drugs that have shown high efficacy in 2D *in vitro* models do not always work or can have a low efficacy in oncology patients (Shoemaker, 2006). This phenomenon is partially explained by the fact that cells grown in 2D cultures do not have a complex three-dimensional tissue architecture and do not exactly reflect the complex interactions between TME or ECM and cells which exist in the body (Figure 1A; Rizvanov et al., 2010).

## Boyden Chamber

The Boyden chamber is a chamber consisting of two compartments filled with medium and separated by a microporous membrane (Falasca et al., 2011). Boyden chamber is a convenient tool for the study of chemotaxis, assessing cell motility and invasion (Figure 1B). Thus, the Boyden chamber was used to assess cell motility in a study on the effect of free paclitaxel and paclitaxel-loaded pyromellitic nanorods on reducing the growth and invasiveness of melanoma cells (Clemente et al., 2019). Wessely et al. (2019) also tested the use of the Boyden chamber to evaluate and compare the invasive activity of spheroids containing only tumor cells and spheroids containing a mixture of tumor and stem cells. Another study examined the adhesion and cytoskeletal migration of HT1080 fibrosarcoma cells and LX2 line stellate cells in a three-dimensional system using fibronectin, Matrigel and type I collagen as chemoattractants (Tovari et al., 2014). However, despite the ease of use of the Boyden chamber, researchers are increasingly turning to more advanced systems that take into account a greater number of TME conditions, in particular, microfluidic systems.

## Three-Dimensional Cultures

It is known that 2D cultures do not fully reflect the pathophysiology of tumor cells and the actual level of resistance to radiotherapy or chemotherapy in the tumor niche in the *in vivo* system (Chen et al., 2012; Table 1). Studies have shown that gene expression profiles as well as treatment responses in multicellular spheroid 3D models are more similar to the *in vivo* situation (Riedl et al., 2017). For example, liver tumor cells in 3D culture have high resistance to drug treatment, similar to the resistance of solid tumors *in vivo* (Uchida et al., 2010). Thus, the BT-549, BT-474, and T-47D breast cancer cell lines cultured as spheroids showed greater resistance to paclitaxel and doxorubicin compared to cells in a 2D culture (Imamura et al., 2015). Cells of squamous cell carcinoma originating from the head and neck (lines LK0902, LK0917, and LK1108) cultured as spheroids were shown to be less sensitivity to cisplatin when compared with 2D cultures. Also in cell lines LK0917 and LK1108, resistance to cetuximab was observed, mediated by culturing in the form of spheroids (Melissaridou et al., 2019). When culturing HCT-116, SW-620, and DLD-1 cells in the form of spheroids or in co-culture with fibroblasts and endothelial cells, their resistance to 5-fluorouracil, regorafenib, and erlotinib preparations increases (Zoetemelk et al., 2019).

**TABLE 1:** Comparative characteristics of cell culture test systems for anticancer drug screening.

**Tumor model**	**Advantages**	**Disadvantages**	**Application**	**Cell type**	**References**
Two-dimensional mono cell cultures	Simple test system for rapid cost effective screening of multiple compounds or libraries	Do not have a complex three-dimensional tissue architecture, complex interactions between TME or ECM and cells	Anticancer drug screening	NCI60 panel	(Shoemaker, 2006)
				JFCR39 panel	(Nakatsu et al., 2007)
Boyden’s chamber	Possibility to study the effect of the test substance on the invasiveness and migration potential of tumor cells	The lack of direct intercellular interactions (the study of paracrine factors only) important for TME	Chemotaxis, assessing cell motility and invasion studies	2D cultures (melanoma, fibrosarcoma and other cell types)	(Tovari et al., 2014; Clemente et al., 2019)
				Spheroids (tumor or tumor and stem cells)	(Kaneda et al., 2019; Wessely et al., 2019)
Microfluidic systems	Can reproduce a specific fluid flow, constant temperature, flow pressure and chemical gradients characteristic of *in vivo* systems	Expensive consumables and equipment, non-standardized protocols	Migration/invasion and extravasation studies	2D cultures (lung adenocarcinoma cells, breast tumor cells and other cell types	(Chen et al., 2010; Wang et al., 2013; Anguiano et al., 2017)
				Co-culture (CAFs + NSCLC cells)	(Yu et al., 2016)
				Breast or liver cancer spheroids	(Yu et al., 2010; Zuchowska et al., 2017)
Three-dimensional spheroids	Can reproduce paracrine and direct intercellular interaction, complex three-dimensional architecture and hypoxic conditions in the center of the spheroid	Do not accurately reproduce interaction between ECM and cells. Difficult to standardize.	Anticancer drug screening, invasion studies	One cell type (breast, liver cancer cells, head and neck squamous cell carcinoma and other cell types)	(Uchida et al., 2010; Imamura et al., 2015; Melissaridou et al., 2019)
				Several cell types (colorectal carcinoma + fibroblasts/endothelial cells)	(Zoetemelk et al., 2019)
Three-dimensional organoids	Accurately reproduce *in vivo* tumor architecture	Difficulty in creating large numbers of homogeneous organoids for high-throughput drug screening	Anticancer drug screening, invasion and extravasation studies	Organoids derived from lung cancer/prostate cancer bone metastasis/bladder cancer tissues	(Kim et al., 2019; Mullenders et al., 2019; Nelson et al., 2020)
				Cerebral glioma/medulloblastoma organoids derived from induced pluripotent stem cells (iPSCs)	(Linkous et al., 2019; Ballabio et al., 2020)
				Colon cancer organoids derived from cancer stem cells (CSCs)	(Otte et al., 2019)
Co-cultures on scaffolds	Complex three-dimensional tissue architecture, complex interactions between TME or ECM and cells	Poor reproducibility and similarity to *in vivo* tumor architecture	Anticancer drug screening, invasion studies, cell infiltration studies	Co-culture of NSCLC cells + fibroblasts + immune cells on Matrigel	(Osswald et al., 2019)
				Co-culture of PDAC cell lines + CAFs surrounding by oligomeric type I collagen	(Puls et al., 2018)
				Co-culture of breast cancer cells + GM637 fibroblasts on reconstitutable tissue matrix scaffold (TMS)	(Rijal and Li, 2017)
3D Bioprinting	Reproducing of complex three-dimensional tissue architecture, mimicking chemical environments in tumor, complex interactions between TME or ECM and cells, possibility to create standardized cellular structures for high-throughput drug screening	Expensive consumables and equipment, low precision of cell positioning	Anticancer drug screening, tumor cell invasion and angiogenesis studies	Co-culture of A549 lung carcinoma cells + HUVEC	(Meng et al., 2019)
				Co-culture of glioblastoma cells and glioblastoma-associated macrophages (GAMs)	(Heinrich et al., 2019)
				Co-culture of breast cancer MCF-7/MDA-MB-468 cells and MCF-12A organoid	(Reid et al., 2019)

It is known that the TME may significantly change the susceptibility of tumor cells to drugs. To solve this problem, new methods were developed for culturing cells using the ECM to model spatial organization, as well as adding various types of cells included in the TME to the culture (Kitaeva et al., 2019). 3D co-cultures of non-small cell lung cancer (NSCLC) and fibroblasts embedded in a Matrigel or encapsulated in alginate are used as models in drug discovery for analysis of immune cell infiltration (Osswald et al., 2019). Also, described is a high-potential tumor spheroid model drug screening, which consists of pancreatic ductal adenocarcinoma (PDAC) cell lines (Panc-1 and BxPC-3) and cancer-associated fibroblasts (CAFs) surrounding by oligomeric type I collagen (Oligomer) for creation of the interstitial ECM supports definition (Puls et al., 2018).

An alternative way to create a novel 3D tumor-tissue model is organoid manner. One of the first of developed spheroid method was a mammospheres, described Dontu et al. (2003). The novel *in vitro* system allowed the propagation of mammary stem and progenitor cells into functional ductal/acinar structures (Dontu et al., 2003). Organoids can be received by two main types of stem cells: pluripotent embryonic stem cells and their synthetic induced pluripotent stem cell counterparts and organ-restricted adult stem cells (Clevers, 2016). Also, organoids received by cultivation small tissue fragments and explants on matrixes or from cultured or sorted cells assembled to organoids *in vitro* (Hu et al., 2018). Organoids from primary lung cancer tissues demonstrated the high reproduction levels of histological and genetic characteristics of *in situ* tissue and their high ability for using them in patient-specific drug trials (Kim et al., 2019). Organoid manner was used for modeling PDAC from patient derived xenografts (PDX) tumors (Nelson et al., 2020) and organoids derived from patient prostate cancer bone metastasis (Lee et al., 2020). Organoids derived from patients with bladder cancer were tested with epirubicin, mitomycin C, gemcitabine, vincristine, doxorubicin, and cisplatin, this model was presented as a prospective model of human bladder cancer (Mullenders et al., 2019; Figure 1C).

## Microfluidic Systems

Microfluidic systems are prospective models for reconstructing the migration, microenvironment, and microcirculation of cells in tumor tissue. Microfluidic systems are small devices that can reproduce a specific fluid flow, constant temperature, fresh medium, flow pressure and chemical gradients characteristic of *in vivo* systems (Ruzycka et al., 2019; Figure 1D).

The microfluidic system using collagen-matrigel hydrogel matrices made it possible to reproduce the microenvironment and experimental conditions for studying the migration and invasion of H1299 lung adenocarcinoma cells. At the same time, Matrigel in low concentrations facilitated the migration of H1299 cells, however, at a high concentration Matrigel slowed the migration of cells, possibly due to their excessive attachment. It has also been shown that the use of antibody-based integrin blockers significantly modulated the mechanisms of H1299 cell migration (Anguiano et al., 2017). A microfluidic system with an incessant supply of nutrient medium through a syringe pump has also been described. It is used to study the effect of the matrix metalloproteinase inhibitor (GM6001) on the formation of invadopodia in A549 lung cancer cells, which is characteristic of cells during invasion (Wang et al., 2013). Microfluidic systems also make it possible to obtain a metastatic model of a tumor, such as breast cancer, which allows the study of antitumor drugs effects on the inhibition of tumor cell migration (Mi et al., 2016). To simulate the extravasation process, a microfluidic system was constructed containing two microfluidic channels and a porous membrane sandwiched between them. The first channel represents the vascular equivalent and contains primary endothelial cells isolated from the pulmonary artery. The second channel acts as a reservoir for collecting migratory tumor cells. In this case, endothelial cells showed *in vivo*-like behavior under flow conditions. The introduced GFP-labeled tumor cells of epithelial or mesenchymal origin were detected using vital imaging, which showed tightly attached tumor cells to the endothelial membrane (Kuhlbach et al., 2018).

## 3D Bioprinting

One of the types of three-dimensional cultures is 3D bioprinting, which enables researchers to create various situations that mimic the processes that occur in the TME (Lee et al., 2016; Truong et al., 2018). 3D bioprinting technology enables the creation of standardized test-systems for screening anticancer drugs (Kingsley et al., 2019; Figure 1E). For example, a model of human hepatoma created using 3D bioprinting was more resistant to an anti-CD147 monoclonal antibody (Metuzumab) than a similar model created on a microfluidic system (Li et al., 2019).

An interesting approach is the combination of several types of cells, tumor and stromal, in a 3D bioprinting model. Breast cancer cells and fibroblasts cultured in 3D bioprinting spheroids as part of an alginate-gelatin hydrogel maintained viability for more than 30 days and were resistant to paclitaxel, which was not observed in 3D bioprinting monocultures of breast cancer cells (Jiang et al., 2018). The trophic role of stromal or immune cells has been shown in other studies. The presence of MSCs in 3D bioprinting hydrogel constructs supported breast cancer cell viability after exposure of doxorubicin (Wang et al., 2018). Application of 3D bioprinting technology also allows immune cell behavior studies in TME. In a 3D bioprinting model, glioblastoma cells were shown to actively recruit macrophages and polarize them in glioblastoma-associated macrophages (GAMs), which in turn contributed to the proliferation and invasiveness of glioblastoma cells (Heinrich et al., 2019). 3D-bioprinting models of breast and pancreatic cancer containing the stromal component (human umbilical vessel epithelial cells (HUVEC), fibroblasts, MSCs) and an ECM analog were described. The resulting 3D bioprinting models repeated the behavior of tumors *in vivo* and *in situ* (Langer et al., 2019).

The using of 3D bioprinting also enables designs that simulate tumor vascularization. 3D organotypic microfluidic platform, integrated with hydrogel biomaterials, were obtained in order to simulate the vascular niche of glioma stem cells (GSCs) obtained from patients (Truong et al., 2018). It has been shown that the microvascular network enhances invasion, supports the proliferation rate and the classic GSCs phenotype (Truong et al., 2018). A three-dimensional model of GSCs is described in the composition of a porous hydrogel containing gelatin, alginate and fibrinogen. GSCs actively proliferated, retained viability and biological properties (nestin expression, differentiation ability) in the resulting 3D bioprinting *in vitro* model, and also had resistance to the cytotoxic effect of temozolomide in contrast to 2D culture. An increase in vascular endothelial growth factor (VEGF) secretion in the first 3 weeks of cultivation was also noted, which indicates the induction of tumor angiogenesis mechanisms (Dai et al., 2016). 3D bioprinting capsules with programmable VEGF and EGF outputs also mimics tumor vascularization. The programmed release of growth factors facilitates control over cell migration and the process of angiogenesis, therefore it is possible to get a dynamic system for the study of metastatic processes (Meng et al., 2019).

Thus, the designs obtained using 3D bioprinting enable us to simulate various processes occurring in TME. Further studies in the field of 3D bioprinting, standardization and validation of the developed tumor models will allow the creation of high-efficiency 3D tumor models in order to obtain new fundamental knowledge about the mechanisms of carcinogenesis and also to more accurately screen potential anticancer drugs and aid individual selection of drugs (Knowlton et al., 2015).

## Conclusion

In recent decades, preclinical trials of antitumor agents have undergone significant changes, in particular, much attention has been focused on the modernization of screening protocols for cell cultures. The widespread use of *in vitro* models in preclinical practice was facilitated by the development of the NCI60 panel. Even after more than 30 years, this model is still actively used for screening anticancer drugs as a reference *in vitro* testing method. However, as knowledge of intercellular interactions within the tumor deepened, as well as the low reliability of testing potential anticancer drugs on the NCI60 panel, in the field of preclinical screening, the need arose to develop more complex and high-tech models. Three-dimensional cultures, representing spheroids and spheroid-like formations grown under various cultivation conditions, partially satisfied this request. Three-dimensional cultures compensated for some of the shortcomings of two-dimensional cultures, in particular those associated with intercellular interactions and interactions with the extracellular scaffold. However, conventional three-dimensional cultures are not quite suitable for assessing the effect of anticancer drugs on important processes as migration, invasion and chemotaxis; such studies require the use of additional devices, for example, chips in microfluidic systems and the Boyden chamber. One of the trends of the last decade has been the use of 3D bioprinting, thanks to which, in theory, it is possible to print fabric with the desired architecture with a sufficiently high resolution. Although at the moment there is no universal protocol for printing this or a standard type of tumor tissue used with it, interest in this technology and the importance of its further development are not weakening. Researchers working in the developing new screening models field may liken the situation to the Greek mythology of Odysseus, finding themselves between Scylla and Charybdis – when the model must be complex enough to take into account most of the microenvironment factors, but at the same time be reproducible, with the ability to correctly interpret the screening results. Existing trends in science, particularly in the field of preclinical screening, are heading precisely toward complicating the models being developed, drawing an analogy, the course for Scylla, which turned out to be a competent choice for Odysseus.

## Author Contributions

VS, KK, and AR conceptualization. KK and VS writing – original draft preparation. VS, CR, and AR writing – review and editing. KK visualization. VS and AR supervision.

## Conflict of Interest

The authors declare that the research was conducted in the absence of any commercial or financial relationships that could be construed as a potential conflict of interest.

## Footnotes

1 https://dtp.cancer.gov/ncialmanac
